# Detection of vascular endothelial growth factor in colon cancer xenografts using bevacizumab based near infrared fluorophore conjugate

**DOI:** 10.1186/1423-0127-21-35

**Published:** 2014-04-29

**Authors:** Bishnuhari Paudyal, Pramila Paudyal, Dilip Shah, Hideyuki Tominaga, Yoshito Tsushima, Keigo Endo

**Affiliations:** 1 Department of Radiology, Thomas Jefferson University, Philadelphia, PA 19107, USA; 2 Department of Diagnostic Radiology and Nuclear Medicine, Gunma University Graduate School of Medicine, Maebashi, Gunma 371-8511, Japan; 3 Center for Translational Medicine, Thomas Jefferson University, Philadelphia, PA 19107, USA; 4 Kyoto College of Medical Science, Nantan, Kyoto 6220041, Japan

**Keywords:** Near infrared fluorescence, VEGF, Optical Imaging, Bevacizumab, Colorectal cancer

## Abstract

**Background:**

The aim of this study was to develop the near infrared fluorescence (NIRF)-based imaging agent for the visualization of vascular endothelial growth factor (VEGF) in colon cancer. AlexaFluor 750 conjugating with bevacizumab, and injected intravenously into nude mice bearing VEGF over-expressing HT29 human colorectal cancer. Optical imaging was performed at 15 min, 24 h and 48 h post injection. Immunofluorescences staining of the tumor sections were performed. HT29 colorectal cancer xenografts were clearly visualized with bevacizumab-AlexaFluor 750.

**Results:**

*Ex vivo* analysis showed 2.1 ± 0.4%, 37.6 ± 6.3% and 38.5 ± 6.2% injected dose/g accumulated in the tumors at 15 min, 24 h and 48 h respectively. Tumor uptake was significantly decreased in pretreated with excess of bevacizumab (p = 0.002). Immunofluorescence analysis showed strong staining of anti-CD 31 antibody around the blood vessels. Anti-VEGF-A and bevacizumab showed heterogeneous expression throughout the tumor.

**Conclusions:**

Current study successfully detected the VEGF expression in HT29 colorectal cancer xenografts, signifying as a potential agent for non-invasive imaging of VEGF expression, which may be applied in clinical practice.

## Background

Molecular imaging has emerged as an indispensable tool in the field of cancer research for *in vivo* monitoring of specific molecular and cellular processes, such as gene expression, multiple simultaneous molecular events, progression or regression of cancer, reliable diagnostic imaging for various malignancy, and it allows for functional assessment of tumors [1-6]. Optical imaging is an emerging modality of choice for preclinical studies to evaluate the expression of different kinds of proteins. It allows visualization of subcellular structures on a microscopic scale, as well as macroscopic distribution of fluorescent labels *in vivo* in small animals [7]. However, high tissue auto fluorescence and limited tissue penetration preclude the use of visible light for most *in vivo* imaging applications. Near infrared (NIR) light solves these problems by reducing fluorescence background and enhancing tissue penetration [8-10].

Over the past several years, there has been an explosion of reports describing successful *in vivo* NIR fluorescence imaging [11-18]. Several optical contrast agents have been developed for the detection of various types of cancer [19-21]. Although most of these studies are qualitative, quantitative studies are beginning to emerge. Vascular endothelial growth factor (VEGF) is upregulated in numerous solid malignancies including colon cancer. VEGF induces and the formation of vesiculo-vacuolar organelles that form channels through which blood-borne proteins can extravasate. This leads to the formation of an extravascular fibrin gel, which provides a matrix that supports the growth of endothelial cells and tumor cells and allows invasion of stromal cells into the developing tumor [22]. Several studies have implicated VEGF in human colon cancer angiogenesis [23]. However, the increased in VEGF expression has been associated with poor prognosis. VEGF overexpression is seen in most cancers, providing an attractive target for molecular therapies [17,24-27]. One of the most successful approaches is the development of bevacizumab, a humanized monoclonal antibody targeting VEGF [28]. Bevacizumab binds with all VEGF-A isoforms and prevents interaction with the VEGF-A receptors, VEGFR-1 and VEGFR-2, and thus inhibits VEGF-mediated angiogenesis [15]. The present study evaluated the feasibility of using NIRF-labeled bevacizumab for tumor imaging in colon cancer xenografts. Bevacizumab was labeled with NIRF agent, and the optical imaging and biodistribution of the conjugate were investigated in nude mice bearing VEGF overexpressing HT29 colorectal cancer xenografts.

## Methods

### Cell lines

Human colorectal cancer cell line HT29 was purchased from American Type Culture (Rockville, USA) and was cultured in McCoy’s medium (Sigma, St Louis, MO, USA) supplemented with 10% fetal bovine serum and antibiotics (100 U/ml penicillin and 100 μg/ml streptomycin).

### Reagents

Bevacizumab (Avastin®, Chugai Pharmaceuticals, Tokyo, Japan) was conjugated with SAIVI AlexaFluor 750, 3 mg labeling kits (Invitrogen, Carlsbad, CA, USA) according to the manufacturer’s protocol. Briefly, 3 mg/ml of the bevacizumab was mixed with sodium bicarbonate and conjugated with Alexa Fluor 750. The purification of labeled antibody was performed in a spin column filled with size exclusion resin in PBS, pH 7.2. Spectrophotometer was used to analyze the moles the stoichiometry of dye-to-protein was estimated from absorbance according to the protocol from Invitrogen. The fluorescent protein was aliquoted and stored in the dark at room temperature.

### Immunofluorescence studies

Frozen tissue slides were fixed in ice-cold acetone and were fixed with 4% paraformaldehyde for 15 min at room temperature, and blocked with 3% BSA in PBS for 1 h at room temperature. The specimens were then incubated with rat anti-mouse CD 31 antibody (PEGAM-1; Becton-Dickinson, Harlingen, CA, USA), anti-mouse VEGF-A (BioLegend, CA, USA), and bevacizumab for 1 h at room temperature. After thoroughly washing with PBS, the slides were visualized with AlexaFluor 488 (Invitrogen, Carlsbad, CA, USA) for 1 h at room temperature. Mounting medium with DAPI (Vector, CA, USA) was used to fix and stain the cell nuclei. The cells were then observed under a fluorescence microscope (Olympus BX50, Tokyo, Japan). Average microvessel density (MVD) was calculated by averaging the number of CD31-positive vessel structures counted from three randomized fields per tumor section at a magnification of 200× under the fluorescence microscope.

### *In vivo* tumor model

Animal experiments were conducted in accordance with Gunma University institutional guideline and were approved by the Animal Care and Use Committee. Female Balb/c nude mice, 6 weeks old (CLEA, Tokyo, Japan) were maintained under specific pathogen-free conditions and were provided with sterile food and water. Nude mice were inoculated subcutaneously with VEGF expressing HT29 cells. When the tumors were 7–8 mm in diameter, the animals were injected intravenously with 50 μg of bevacizumab-AlexaFluor 750. For competitive blocking studies, another group of mice (n = 4) were pretreated with excess fold of unconjugated bevacizumab before administering the fluorescently labeled bevacizumab. Blood was withdrawn from the tail veins of xenografted mice to determine the concentration of VEGF in serum. Mice were examined by optical imaging.

### Fluorescence imaging

The *in vivo* optical imaging was carried out with a small animal *in vivo* imaging system (Maestro CRI, MA, USA). The animals were anesthetized with 10% pentobarbital sodium before the acquisition was started. A customized filter set (excitation, 710–760 nm; emission, 695–800 nm) was used for NIRF data acquisition. A group of 4 mice were used at each time point for the experiments. The biodistribution of fluorescence intensity was monitored at 15 min, 24 h and 48 h post-injection. In order to quantitatively estimate the accumulation of the probe in selected organs, animals were killed by decapitation. Tissues of interest were excised and weighed. The fluorescence intensity of each tissue was measured and normalized to photons per second with an ROI covering the entire tissue. The total fluorescence flux of each tissue was divided by its weight. The results were calculated as % injected dose/gram (% ID/g).

### Statistical analysis

Data were expressed as mean ± standard deviation. Means were compared using unpaired student’s *t*-test. *P* values of less than 0.05 were considered statistically significant.

## Results

### Synthesis and characterization of bevacizumab-AlexaFluor 750

The molar ratio of AlexaFluor 750 to bevacizumab was determined to be 2:1. The absorption and fluorescence emission characteristics of the bevacizumab-AlexaFluor 750 was found to be similar to those of free AlexaFluor 750, as apparent from the spectra measured in PBS, suggesting that the fluorescence property of the AlexaFluor 750 was not affected by the conjugation to the bevacizumab.

### *In vivo* imaging studies with bevacizumab-AlexaFluor 750

**Figure 1:**
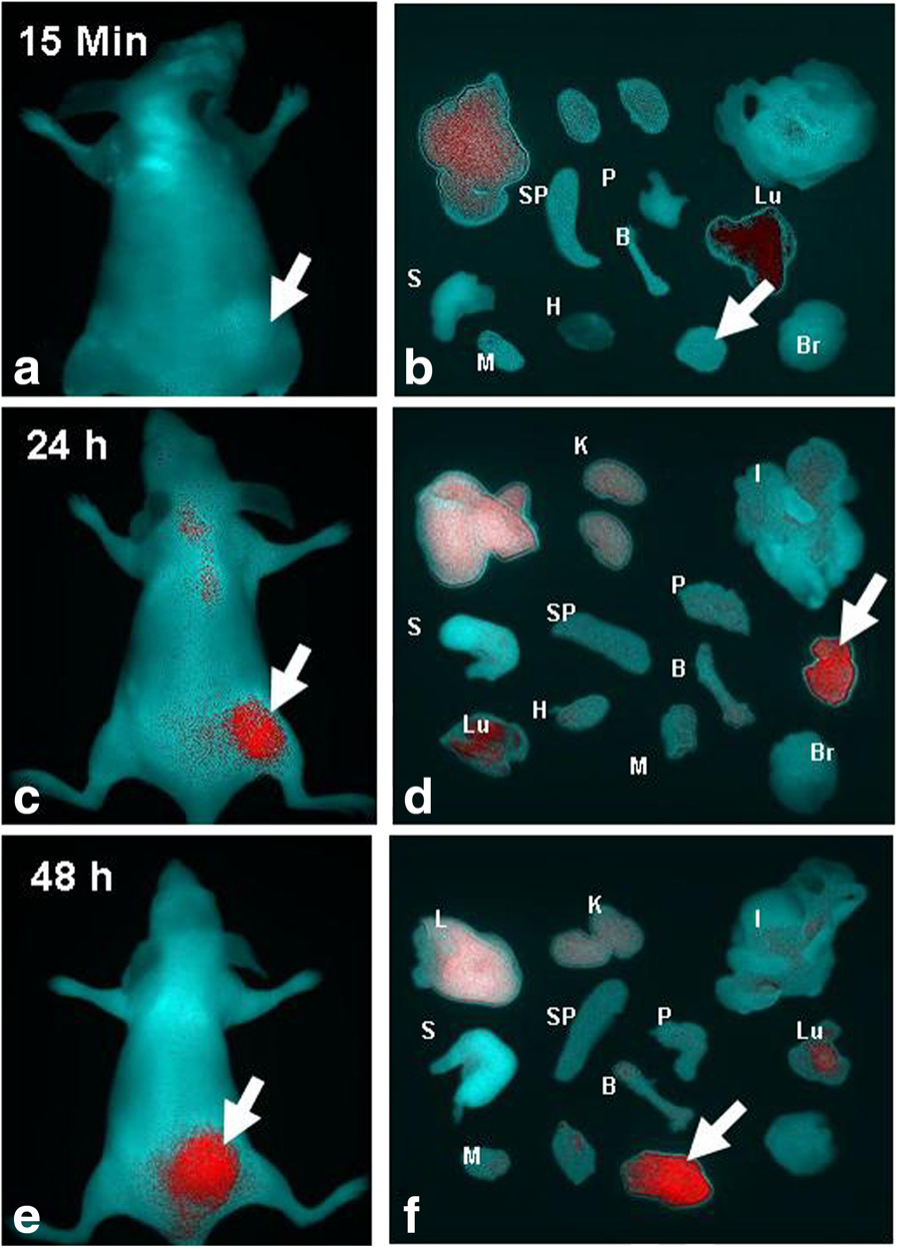
**NIRF images of HT29 human colon cancer bearing mice obtained at 15 min (a-b), 24 h (c-d) and 48 h (e-f) after intravenous injection of bevacizumab-AlexaFluor 750.***Arrows* indicate tumor, L: Liver, I: Intestine, S: Stomach, Sp: Spleen, P: Pancreas, Lu: Lung, H: Heart, M: Muscle, B: Bone, Br: Brain.

**Figure 2:**
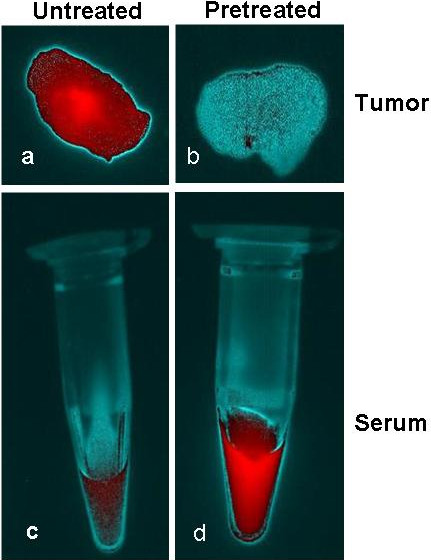
**The tumor and serum (the representative HT29 tumor bearing mouse) was pretreated with excess fold of bevacizumab: tumor VEGF (a-b) and serum VEGF (c-d).** Tumor VEGF in the pretreated group of mice is significantly (*p* = 0.002) suppressed **(b)** in the accumulation of bevacizumab-AlexaFluor 750 in comparing with untreated tumor Figure 2**(a)**, while the serum VEGF is relatively higher **(d)** comparing to the untreated serum **(c)**.

**Figure 3:**
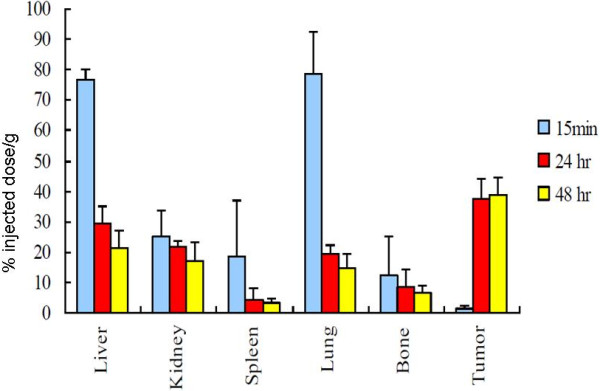
**Biodistribution studies of bevacizumab-AlexaFluor 750 in HT29, human colon cancer bearing mice at 15 min, 24 h and 48 h.** Data compare in percent injected dose per gram in the organs after injection with bevacizumab-AlexaFluor 750. Tumor uptake reached the peak at 24 h.

Figure 1 shows NIR fluorescence images of mice bearing VEGF positive HT29 tumors at 15 min (a-b), 24 h (c-d), and 48 h (e-f). *In vivo* fluorescence detection revealed distinct uptake in the VEGF overexpressing HT29 tumors at 24 h and 48 h after the IV injection of bevacizumab-AlexaFluor 750. The fluorescence signal was clearly visualized in the tumor area as compared with normal regions. The HT29 tumor bearing xenografts pretreated with excess fold of bevacizumab Figure 2(b) showed a significant (*p* = 0.002) decrease in the accumulation of bevacizumab-AlexaFluor 750 in comparing with untreated tumor Figure 2(a), illustrating the specificity that completely block the bevacizumab-Alexa Fluor750. Quantitative analysis of the *ex vivo* tissue fluorescence images post-injection with bevacizumab-AlexaFluor 750 (Figure 3) demonstrates the progressive accumulation of bevacizumab-AlexaFluor 750 by the VEGF positive HT29 tumors (2.1 ± 0.4% ID/g), (37.6 ± 6.3% ID/g) and (38.5 ± 6.2% ID/g) at 15 min, 24 h and 48 h respectively, post injection. The tumor to blood ratio was 1.3 ± 1.1 at 24 h. and 1.5 ± 1.2 at 48 h. Intense fluorescence was seen in the blood and blood-rich organs like lung (78.5 ± 13.8% ID/g) and liver (76.8 ± 3.0% ID/g) at 15 min, which decreased at 24 h to 19.2 ± 3.0% and 48 h to 29.6 ± 5.3% ID/g, respectively (Figure 3). The fluorescence intensity in muscle and other non-target regions were nominal.

### Immunofluorescence studies

**Figure 4:**
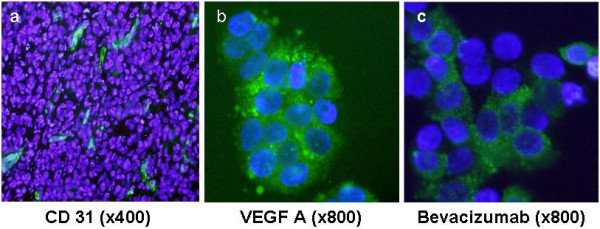
Immunofluorescence staining of HT29 tumor with: (a) anti-CD 31 antibody (400×), (b) anti-VEGF-A antibody (800×), and (c) bevacizumab (800×) in frozen sections (Green: antibody labeled AlexaFluor 488, blue is DAPI).

**Figure 5:**
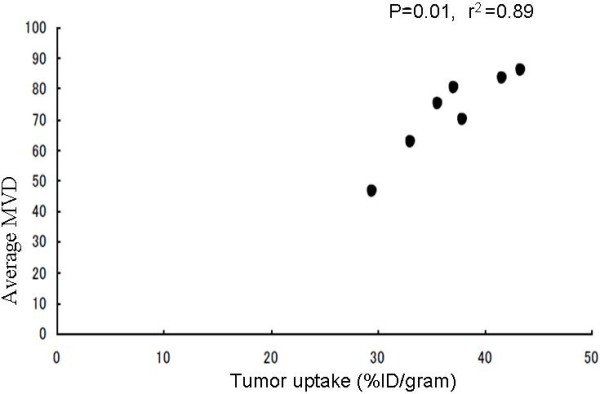
**Significant correlation is noted (*p*** **= 0.01, r**^**2**^ **= 0.89), between the presences of average micro vessel density in three random fields and the tumor uptake as measured by % ID/g.**

To characterize the localization of VEGF, histological analysis of the tumor specimens was performed using anti-CD31, anti-VEGF-A, and bevacizumab. Figure 4(a-c) shows the expression of CD 31 and VEGF. Strong staining by the anti-CD31 antibody is noted around the blood vessels formed within the tumor section. The expression pattern of VEGF was heterogeneous throughout the cytoplasm. Both bevacizumab and anti-VEGF-A showed similar cytoplasmic staining, suggesting the binding of both antibodies to the VEGF antigen. A significant correlation was obtained between the bevacizumab-AlexaFluor 750 % ID/g uptake of the tumors and their respective MVD (*p* = 0.01, r^2^ = 0.89), as shown in Figure 5.

## Discussion

The present study reports the development and validation of the bevacizumab-AlexaFluor 750 conjugates as a NIRF imaging agent for noninvasive imaging of VEGF expression in human colon cancer xenografts in mice. *In vivo* assessment of VEGF within a tumor using bevacizumab-AlexaFluor 750 offers further information about the angiogenic activity of the tumor.

Here we showed a significant VEGF specific accumulation of bevacizumab-AlexaFluor 750 in the tumor region. Immunofluorescence microscopy with anti-VEGF-A and bevacizumab showed heterogeneous expression throughout the cytoplasm of HT29 cells. The presence of adequate microvessels was noted when the slides were incubated with anti-CD31 antibody, which might have contributed to the higher accumulation of the bevacizumab-AlexaFluor 750. A significant linear correlation was noted between the MVD and the tumor accumulation as determined by % ID/g, in agreement with the role of VEGF in angiogenesis. The mice treated with excess folds of bevacizumab before the administration of bevacizumab-AlexaFluor 750 showed decreases in serum than those without pretreatment however the difference was not significant. This discrepancy may be due to the fact that, even though the energy absorbed due to water or other naturally occurring fluorochromes is low at the near infrared window, the sensitivity is not as high as it is in the case of hemoglobin, suggesting its limited use as a primary imaging modality.

Generally, serum VEGF is measured to monitor the VEGF expression before the initiation of therapy however VEGF concentration determined from circulation are invasive and are obscure due to the fact that circulating VEGF not only measures the tumor VEGF but also the VEGF secreted by platelets, granulocytes, monocytes, mast cells, and lymphocytes which might give a false positive result [29]. Understanding the complex role of VEGF in tumor development has been somewhat limited by the absence of methods to measure this cytokine spatially and temporally [30]. There is a need for the development of noninvasive imaging to allow monitoring of angiogenesis related molecular events. Reliable markers that can predict which patients are more likely to respond to anti-VEGF therapy would be important, but they have been elusive so far.

Few studies have documented the ability to image VEGF *in vivo* using radiolabeled bevacizumab [31-34]. Recently, Scheer *et al.* found no correlation between the level of antibody accumulation and expression of VEGF, measured by ELISA in the liver metastasis [35]. Even though these studies possess useful information on the diagnosis, imaging with radiolabeled compounds requires expensive equipment and exposes patients to ionizing radiation. Optical imaging is emerging as a sensitive, specific, inexpensive, and convenient technology that permits the study of a variety of cellular and molecular processes *in vivo*. Earlier studies reported that NIR fluorophores can be detected 7–14 cm deep in tissue [9]. Nevertheless, it suffers from poor detectability due to low tissue penetration. This inspection, together with our results and the continued development of versatile imaging devices, could overcome these issues and therefore make optical imaging the modality of choice.

In clinical settings, optical imaging is relevant for tissues close to the surface of the skin, tissues accessible by endoscopy, and intraoperative visualization. Multimodality imaging approach can be developed such as further labeling of bevacizumab-AlexaFluor 750 with radionuclides for PET and SPECT as well as a contrast agent for MRI to enhance the sensitivity and specificity of biomedical imaging *in vivo.*

The large size of an antibody impacts its ability to move through a tumor mass. A high interstitial pressure inhibits the diffusion of larger molecules within the tumor migration within the tumor is also inhibited by a binding site barrier [35-37]. Administering higher doses of the antibody can reduce the effect of the binding site barrier and allow the antibody to diffuse more deeply into the tumor bed [37]. The present study showed that the tumor uptake of bevacizumab-AlexaFluor 750 in HT29 xenografts was high, and lower in the non-targeted tissues. The high tumor accumulation is the most important property of the optical imaging tracer. These results warrant the further assessment of this bevacizumab-AlexaFluor 750 for imaging in colon cancer and other tumor models.

## Conclusions

In conclusion, we could successfully detect the VEGF expressing tumors using bevacizumab-AlexaFluor 750 *in vivo*. Because the majority of tumor vasculature overexpresses VEGF during angiogenesis, bevacizumab-AlexaFluor 750 has great potential for detecting tumor vasculature *in vivo* in general for most cancer types. The results reported here open up new perspectives for VEGF-targeted imaging in a less expensive way.

## Competing interests

The authors declare that they have no competing interests.

## Authors’ contributions

BP designed, performed research, analyzed the data, reviewed the paper and wrote the manuscript. PP performed research, analyzed the data, and wrote manuscript. DS prepared figures, reviewed the paper. HT performed research. YT and KE analyzed the data and reviewed the paper. All authors read and approved the final manuscript.
